# Foot Drop Caused by Lumbar Degenerative Disease: Clinical Features, Prognostic Factors of Surgical Outcome and Clinical Stage

**DOI:** 10.1371/journal.pone.0080375

**Published:** 2013-11-05

**Authors:** Kun Liu, Wei Zhu, Jiangang Shi, Lianshun Jia, Guodong Shi, Yuan Wang, Ning Liu

**Affiliations:** 1 Department of Orthopedic Surgery, the Second Artillery General Hospital, Beijing, China; 2 Department of Orthopedic Surgery, Shanghai Chang Zheng Hospital, Second Military Medical University, Shanghai, China; University of Louisville, United States of America

## Abstract

**Objective:**

The purpose of this study was to analyze the clinical features and prognostic factors of surgical outcome of foot drop caused by lumbar degenerative disease and put forward the clinical stage.

**Methods:**

We retrospectively reviewed 135 patients with foot drop due to lumbar degenerative disease. The clinical features and mechanism were analyzed. Age, sex, duration of palsy, preoperative muscle strength of tibialis anterior (TA), sensation defect of affected lower limb, affected foot, diagnosis and compressed nerve roots were recorded and compared with surgical outcome.

**Results:**

Foot drop was observed in 8.1% of all inpatients of lumbar degenerative disease. L5 nerve root compression was observed in 126 of all 135 patients (93.3%). Single, double and triple roots compression was observed respectively in 43, 83, and 9 patients (31.9%, 61.5%, and 6.6%). But there was no significant relationship between preoperative muscle strength of TA and the number of compressed roots. The muscle strength of TA was improved in 113 (83.7%) patients after surgery, but it reached to >=4 in only 21 (15.6%) patients. Improvement of the muscle strength of TA was almost stable at the 6-month follow-up. At the last follow-up, the muscle strength of TA was 1, 2, 3, 4, 5 respectively in 28, 24, 62, 13, 8 patients. Multivariate logistic regression showed duration of palsy (p=0.0360, OR=2.543), preoperative muscle strength of TA (p=0.0064, OR=5.528) and age (p=0.0309, OR=3.208) were factors that influenced recovery following an operation.

**Conclusions:**

L5 nerve root was most frequently affected. The muscle strength of TA improved in most patients after surgery, but few patients can get a good recovery from foot drop. Patients of shorter duration of palsy, better preoperative muscle strength of TA and younger age showed a better surgical outcome.

## Introduction

Foot drop is characterized by the inability or difficulty in moving the ankle and toes upward (dorsiflexion). It is a sign of an underlying neurological, muscular or anatomical problem. Most of the time, foot drop is the result of neurological disorder; only rarely is the muscle diseased or nonfunctional. The source for the neurological impairment can be central (motor neuron disease, parasagittal cortical or subcortical cerebral lesions) or peripheral (lumbar radiculopathy, mononeuropathies of the deep peroneal, common peroneal, or sciatic nerves) [1-3]. In addition, there have been several case reports of foot drop due to an unusual etiology such as Baker's cyst [4], Hodgkin's lymphoma [5], Perineurioma [6], Paget's disease [7] and iatrogenic foot drop after spinal anesthesia [8] or lumbar surgery [9,10].

Lumbar degenerative disease (LDD) including lumbar disc herniation (LDH) and lumbar spinal stenosis (LSS) is a common etiology for low back pain and leg pain. LDD is frequently seen in elderly patients and can results in severe symptom, such as intermittent claudication and sensation defect of lower limb. However foot drop caused by LDD is relatively rare. And few studies have addressed this phenomenon [11-14].

In the current study, we retrospectively reviewed 135 patients who had foot drop caused by LDH and/or LSS and underwent lumbar surgery. We analyzed the clinical features and mechanism. Several potential prognosis factors were recorded and compared with surgical outcome. Based on the research, we put forward the clinical stage for foot drop caused by lumbar degenerative disease.

## Materials and Methods

### Patient Selection

The study was reviewed and approved by the Ethics Committee of Chang Zheng hospital, Shanghai, China. Written informed consent was obtained from each patient. This consent procedure was also approved by the Ethics Committee. The study cases were selected from 1674 patients suffered LDH and/or LSS and underwent surgery by the same senior surgeon (L.J.) and his colleagues in our department from January 2005 to January 2010. Patients who had other spinal deformity diseases (such as scoliosis), fracture or underwent spine surgery before were excluded. 135 patients who presented foot drop were assessed in the current study. The incidence of foot drop was 8.1% in patients of LDD. Of the patients, there were 62 men and 73 women, the mean age at surgery was 55 years (range 43-64), the mean duration of foot drop was 186.4 days (range 14-365), the mean of follow-up period was 2.3 years (range 2-3).

Tibialis anterior (TA) was designed to pick up the foot, palsy of this muscle resulted in inability or difficulty in moving the ankle and dorsiflexion. Therefore foot drop was defined as muscle strength of TA using a manual muscle test (MMT) below or equal to 3 (out of 5) in the current study [15]. And it was unilateral in 117 patients (74 right, 43 left) and bilateral in 18 patients. When foot drop was bilateral, the weaker one was used for grading. All of the patients suffered low back pain and leg pain before foot drop. 93 patients suffered a sensation defect of affected lower limb.

The muscle strength of TA was scored on MMT after hospitalization. It was 1.66 on average, and 0, 1, 2, 3 respectively in 18, 34, 59, 24 patients.

Diagnoses were established based on history and physical examination in conjunction with MRI and/or CT findings. Sixty-two (45.9%) patients were diagnosed as isolated LDH, and 73 (54.1%) had LSS (canal size < 75% of that in the nonstenotic segments, and/or lateral stenosis) with associated various degree of disc herniation. 

The affected vertebral levels (disc herniation and/or spinal canal stenosis) were determined according to the findings of MRI and CT, and surgery was operated on these levels. The compressed nerve roots showed on MRI were confirmed during operation.

### Surgical Technique

**Figure 1:**
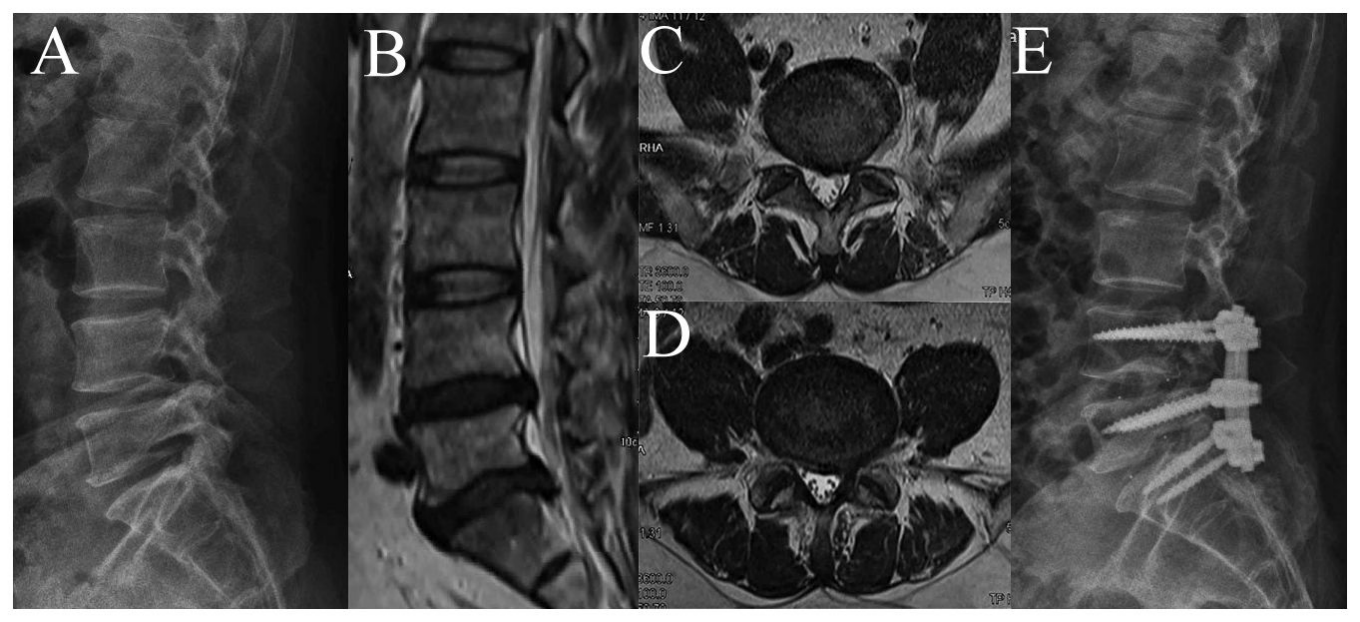
A 54-years-old man, diagnosed as LDH and left foot drop. (A) Preoperative radiography. (B) Preoperative mid-sagittal MRI showed LDH on L4-S1. (C) Preoperative axial MRI of L4-5 showed the left L5 nerve root compression. (D) Preoperative axial MRI of L5-S1 showed the left S1 nerve root compression. (E) Postoperative radiography.

All patients underwent nerve roots decompression and modified posterior lumbar interbody fusion (PLIF) with pedicle screw instrumentation by the same senior surgeon and coworkers (Figure 1). Under general anaesthesia, patients were placed prone. Bilateral pedicle screw fixation was performed on the affected levels (MOSS MiamiTM, Depuy Spine, Johnson & Johnson, USA). After hemilaminectomy of parts of inferior and superior laminae, intervertebral disks were removed from one side. And nerve roots decompression was performed unilateral or bilateral by removing degenerated ligamenta flava, articular process, lamina of vertebra and protruded nucleus pulposus. There was a potential instability of lumbar spine because of resection of parts of articular process. So Cages were implanted unilaterally for fusion (SABER I/F CAGE^TM^, Depuy Spine, Johnson & Johnson, USA). The intraoperative bleeding was controlled under 800ml, and 24 patients was transfused blood component. There were no serious postoperative complications.

### Postoperative Follow-Up

Patients stayed in hospital for 3 to 7 days after surgery. Visual Analogue Scale (VAS) score system was used to measure pre and postoperative low back pain and leg pain. The muscle strength of TA was examined on MMT in 2 weeks, 1 month, 3 months, 6 months, 1 year and 2 years until it was stable. When the muscle strength of TA recovered to 4 or 5, patients could move the ankle and reached a relative normal gait, which is considered as recovery; and the remaining was unrecovered patients. The recovered patients were divided into two groups further: completely recovered patients (muscle strength of TA =5) and incompletely recovered patients (muscle strength of TA =4).

### Statistical Methods

Clinical characteristics of patients between group LDH and group LSS were compared. We also analyzed the duration of foot drop before operation, preoperative muscle strength of TA, age, sex, sensation defect of affected lower limb, affected foot, diagnosis and compressed nerve roots to determine which is the prognosis factor comparing the recovered with unrecovered patients. The statistical analysis was carried out using a Mann-Whitney U test and Fisher exact probability test. To analyze the synergistic effect of multiple factors which may influence surgical outcome, multivariate logistic regression analysis using stepwise selection was performed (sle= sls =0.15, age and duration of palsy were stratified by respectively 5 years and 30 days). Probability values <0.05 were considered to be statistically significant. We used SPSS (Version 16.0; SPSS Inc, Chicago, IL, USA) for all analyses.

## Results

Based on the result of MRI and CT, 9 patients were three levels affected (L3-4 and L4-5 and L5-S1), 83 patients were two levels affected (L4-5 and L5-S1 in 56 patients, L3-4 and L4-5 in 27), and 43 patients were one level affected (L3-4, L4-5, L5-S1 respectively in 2, 34, 7).

**Figure 2:**
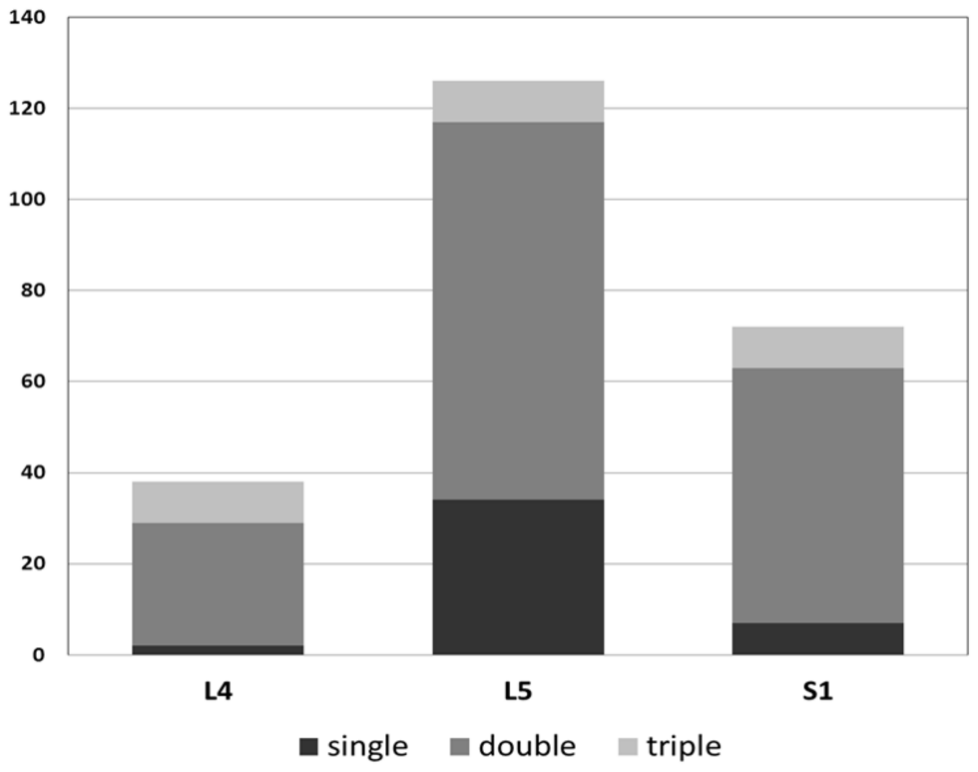
Compressed nerve roots in patients with foot drop. L5 nerve root was most frequently affected. Double or triple roots compression was a common condition.

On the basis of operative finding, L5 and S1 nerve roots compression was the most common condition (56 patients, 41.5%), followed by L5 (34 patients, 25.2%), L4 and L5 (27 patients, 20.0%), L4 and L5 and S1 (9 patients, 6.6%), S1 (7 patients, 5.2%) and L4 (2 patients, 1.5%). And L5 was the most frequently affected nerve root observed (126 patients, 93.3%), followed by S1 (72, 53.3%) and L4 (38, 28.1%) (Figure 2). There was no significant relationship between muscle strength of TA and the number of compressed roots (Pearson correlation test, p=0.2693).

Clinical characteristics of patients in group LDH and group LSS were provided in Table 1. Patients in group LDH were significantly younger than patients in group LSS (p=0.0015). And the number of compressed nerve roots of patients in group LDH was significantly smaller than that in group LSS (p<0.0001).

**Table 1:** Clinical characteristics of patients in group LDH and group LSS.

Factor	LDH (62)	LSS (73)	p value
mean preop Sx duration (days)	192.5	181.2	0.3291*
mean preop muscle strength of TA	1.75	1.58	0.1836*
mean age (yrs)	44.7	63.7	0.0015*
sex (M/F)	28:34	34:39	1†
sensation defect of lower limb (yes/no)	38:24	55:18	0.0945†
affected foot (bilateral/unilateral)	8:54	10:63	1†
compressed nerve roots (multiple/single)	25:37	67:6	<0.0001†
outcome (recovered/unrecovered)	13:49	8:65	0.1525*

LDH= lumbar disc herniation; LSS= lumbar spinal stenosis; TA= tibialis anterior; * Mann-Whitney U-test; † Fisher exact probability test

All patients had an obvious release from low back pain and leg pain after surgery. The mean preoperative VAS score for low back pain and leg pain was 5.4 (range, 4-8), and it was 2.2 (range, 0-4) at the 2-week follow-up. And the sensation of affected lower limb of the 93 patients was improved too.

The muscle strength of TA at different follow-up period was showed in Table 2, and the result at the last follow-up was showed in Table 3. The muscle strength of TA improved in 113 patients (83.7%), compared with 22 patients unimproved (16.3%). The muscle strength of TA recovered to 4 or 5 in 21 of 135 patients (15.6%) (recovered patients), and it recovered to ≤ 3 in the remaining 114 patients (unrecovered patients). With regard to the 21 recovered patients, the muscle strength of TA recovered to 5 in 8 patients (38.1%), and 4 in 13 (61.9%). 

**Table 2:** The improvement status of muscle strength of tibialis anterior at different period of follow-up.

Time / Number	Muscle strength of tibialis anterior					
	0	1	2	3	4	5
Preoperation	18	34	59	24	0	0
2-week follow-up	12	23	60	37	3	0
3-month follow-up	0	28	24	71	10	2
6-month follow-up	0	28	24	62	15	6
1-year follow-up	0	28	24	62	13	8
2-year follow-up	0	28	24	62	13	8

**Table 3:** The improvement status of muscle strength of tibialis anterior at the last follow-up.

Preop muscle strength of TA	Muscle strength of TA at the last follow-up						
	Number	0	1	2	3	4	5
0	18	0	18	0	0	0	0
1	34	0	10	20	4	0	0
2	59	0	0	4	50	3	2
3	24	0	0	0	8	10	6

TA= tibialis anterior

In a comparison of each factor (duration of symptoms before operation, preoperative muscle strength of TA, age, sex, sensation defect of affected lower limb, affected foot, diagnosis and compressed nerve roots) between the recovered and unrecovered patients, duration of palsy (p= 0.0362), preoperative muscle strength of TA (p=0.0025) and age (p= 0.0213) were found to have a significant influence on the prognosis (Table 4). And multivariate logistic regression analysis showed the same result (duration of palsy: OR= 2.543, p= 0.0360; preoperative muscle strength of TA: OR=5.528, p=0.0064; age: OR= 3.208, p=0.0309; Table 5).

**Table 4:** Factor comparison for patients of lumbar degenerative disease stratified by recovery status.

Factor	Recovered (21)	Unrecovered (114)	p value
mean preop Sx duration (days)	86.3	204.9	0.0362*
mean preop muscle strength of TA	2.55	1.83	0.0025*
mean age (yrs)	49.7	56.0	0.0213*
sex (M/F)	11:10	51:63	0.6350†
sensation defect of lower limb (yes/no)	12:9	81:33	0.2111†
affected foot (bilateral/unilateral)	3:18	15:99	1†
dignosis (LDH/LSS)	13:8	49:65	0.1525*
compressed nerve roots (multiple/single)	19:2	107:7	0.6302†

TA= tibialis anterior; LDH= lumbar disc herniation; LSS= lumbar spinal stenosis; * Mann-Whitney U-test; † Fisher exact probability test

**Table 5:** Multivariate Logistic Regression for patients of lumbar degenerative disease stratified by recovery status.

Factor	p value	Odds Ratio	95% CI
Preop Sx duration (per 30 days)	0.0360*	2.543	1.063-6.083
Preop muscle strength of TA	0.0064*	5.528	3.759-6.842
Age (per 5 yrs)	0.0309*	3.208	1.113-9.240

TA= tibialis anterior; CI= confidence interval; * Multivariate Logistic Regression using stepwise selection, sle= sls= 0.15

Based on the research, we put forward the clinical stage of foot drop caused by lumbar degenerative disease. That is early stage, middle stage, and late stage (Table 6). 

**Table 6:** Clinical stage of foot drop caused by lumbar degenerative disease.

Factor	Weight						
	0	1	2	3	4	5	6
Duration of palsy (days)	>180	150-180	120-150	90-120	60-90	30-60	<30
Preop muscle strength of TA	0	1	2	3			
Age (yrs)	>60	55-60	50-55	45-50	<45		

TA= tibialis anterior

Early Stage: 10-13, Good recovery

Middle Stage: 5-9, Incomplete recovery

Late Stage: 0-4, Poor recovery

## Discussion

This is a relative large sample (135) retrospective study. The strengths of this study are that the clinical and imaging data provides insights into the clinical features of foot drop caused by lumbar degenerative disease, and nearly all known factors were included in the analysis to determine which factor influenced surgical outcome. The present study revealed that L5 nerve root was most frequently affected. The muscle strength of TA was improved in 113 (83.7%) patients after surgery, but it reached to >=4 in only 21 (15.6%) patients. The analysis showed patients of shorter duration of foot drop, better preoperative muscle strength of TA and younger age had a better recovery. Furthermore, we put forward clinical stage of foot drop caused by lumbar degenerative disease based on the statistical analysis.

There have been several studies addressing the mechanism of foot drop caused by lumbar degenerative disease. But data is still limited, and there are some controversies.

It has been reported that TA is innervated and controlled by L4 root in several anatomical studies [10,16]. However, Aono reported that the main lesion occurred in the L4-5 segment, and concluded that foot drop would be mainly caused by an impairment of the L5 nerve root and this root mainly ruled TA [11]. McCulloch showed that the L5 root innervated TA and extensor hallucis longus but adjacent roots (L4 and S1) also innervated those muscles, based on the results of electrical stimulation studies [17]. And the study of Iizuka showed that an impairment of double nerve roots including the L5 root is a common mechanism of foot drop caused by lumbar disc herniation [14]. In the current study, L5 and S1 roots compression was the most common condition (56 patients, 41.5%), L5 root was the most frequently affected nerve root observed (126 patients, 93.3%), but only L4 or S1 root compression was also observed in 2 or 7 patients respectively. We concluded that L5 root mainly innervated TA, in addition, L4 and S1 root also innervated this muscle, which supported the research of McCulloch [17]. The complexity of compressed nerve roots suggests that the common peroneal nerve which controls TA contains the fibers from L4-S1 roots. Further anatomical and electro-neurophysiology studies are necessary.

Several studies showed surgery was an effective method to treat foot drop caused by lumbar degenerative disease, although motor function of spinal roots were thought hardly recovered after damage traditionally [11-14]. In the study of Aono, 61% of patients recovered from drop foot after surgery (muscle strength of TA >=4 on MMT), of all 46 patients, 14 patients had complete recovery (muscle strength of TA =5 on MMT), and 13 (28.3%) had no improvement after operation [11]. Similarly, Iizuka showed the muscle strength of TA recovered to 4 or 5 in 12 of 16 patients suffered herniated nucleus pulposus and 3 of 12 patients suffered lumbar spinal stenosis [14]. In his study, the postoperative muscle recovery in patients with herniated nucleus pulposus was significantly superior to that in patients with lumbar spinal stenosis. In the current study, the muscle strength of TA improved in 113 patients (83.7%) after surgery, but only 15.6% of patients recovered from foot drop and 5.9% of patients had a complete recovery. Surgery of nerve roots decompression is beneficial to patients of foot drop. However this study showed few patients can get a satisfying recovery.

The prognosis factors of foot drop due to lumbar degenerative disease had been reported in several studies, but there are still some controversies. A study of 55 patients showed no statistically significant relationship was found between the extent of recovery and age, diagnosis (herniated nucleus pulposus *vs.* lumbar spinal stenosis), duration of symptoms, or severity of preoperative weakness [13]. In contrast, Aono showed palsy duration and preoperative strength were factors that most affected drop foot recovery following surgical intervention for spinal degeneration in a study of 46 patients [11]. And Iizuka showed there was no prognostic factor in surgically treated herniated nucleus pulposus (16 patients), but significant associations with prognosis were observed with respect to preoperative muscle strength of TA and extensor hallucis longus in patients with lumbar spinal stenosis (12 patients) [14]. However, in the study of Ghahreman (56 patients), acute disc prolapse was the compressive pathology in 88%; and analysis showed younger patients made a better recovery, no other significant associations between the demographic or clinical features and the recovery of the weakness could be identified [12]. There were 135 patients suffered LDH and/or LSS in the current study, the analysis showed patients of shorter duration of foot drop, better preoperative muscle strength of TA and younger age had a better recovery. Studies showed different or even contrary results regarding to the prognosis factors, due to the limitation of study sample and patients selection of different etiology. Therefore, more studies of large sample and mechanism researches are essential to obtain more reliable evidence.

This clinical stage was based on our statistical analysis. The stage criteria contained duration of palsy, preoperative muscle strength of TA and age. Young patients may have a better and faster recovery. And according to our study and experience, TA would become atrophic and difficult to recover if it was paralytic for a long duration. So duration of palsy and preoperative muscle strength of TA were significant prognosis factors theoretically. For an ideal stage, the quantification of criteria is crucial and necessary. However, it is controversial for prognosis factors of surgical outcome, and it is difficult to draw definitive conclusions from this data set of 135 patients.

In conclusion, foot drop caused by lumbar degenerative disease was often unilateral. L5 nerve root was most frequently affected. Double or triple roots compression was a common condition. The muscle strength of TA improved in most patients after surgery, but few patients can get a good recovery from foot drop. Duration of palsy, preoperative muscle strength of TA and age were factors that influenced recovery following an operation.
